# Ratio of lymph node to primary tumor SUV_max_ multiplied by maximal tumor diameter on positron emission tomography/integrated computed tomography may be a predictor of mediastinal lymph node malignancy in lung cancer

**DOI:** 10.1097/MD.0000000000005457

**Published:** 2016-11-18

**Authors:** Yi Liu, Yanhua Tang, Zhiqiang Xue, Ping Yang, Kefeng Ma, Guangyu Ma, Xiangyang Chu

**Affiliations:** a Department of Thoracic surgery, The Chinese People's Liberation Army (PLA) General Hospital; b Department of Radiology, Beijing Chao-Yang Hospital; c Department of Nuclear medicine, Chinese PLA General Hospital, Beijing, China; d Department of Health Sciences Research, Division of Epidemiology, Mayo Clinic College of Medicine, Rochester, MN.

**Keywords:** diagnosis, lung neoplasms, mediastinal lymph node, neoplasm staging, positron emission tomography

## Abstract

Positron emission tomography/integrated computed tomography (PET/CT) provides the most accurate imaging modality for preoperative lung cancer staging. However, the diagnostic accuracy of maximum standardized uptake value (SUV_max_) for mediastinal (N2) lymph nodes (LN) is unclear. We compared SUV_max_, the ratio of LN to primary tumor SUV_max_ (SUV_n/t_), and SUV_n/t_ multiplied by maximal tumor diameter (SUV_index_) in terms of their abilities to predict mediastinal LN malignancy.

We retrospectively analyzed 170 mediastinal LN stations from 73 consecutive patients who underwent systemic LN resection and PET/CT within 27 days. The SUV_max_ of the primary tumors was >2.0 and the SUV_max_ of the mediastinal LN stations ranged from 2.0 to 7.0 on PET/CT. Receiver-operating characteristic curves (ROCs) of SUV_max_, SUV_n/t_, and SUV_index_ were calculated separately and the areas under the curves (AUCs) were used to assess the abilities of the parameters to predict LN malignancy. The optimal cutoff values were calculated from each ROC curve and the diagnostic abilities were also compared. The diagnostic accuracies of the 3 methods were also assessed separately in smoking and nonsmoking patients.

Twenty-eight LN stations were malignancy-positive and the remaining 142 were malignancy-negative. The AUCs for SUV_index_, SUV_n/t_, and SUV_max_ were 0.709, 0.590, and 0.673, respectively, and the optimal cutoff values for SUV_index_, SUV_n/t_, and SUV_max_ were 1.11, 0.34, and 3.6, respectively. The differences between SUV_index_ and SUV_n/t_ were significant, but there was no significant difference between SUV_index_ and SUV_max_. There were no significant differences between smokers and nonsmokers in the AUCs for any of the methods for predicting LN malignancy (*P* values >0.05).

SUV_index_ may be a predictor of mediastinal LN malignancy in lung cancer patients.

## 1 Introduction

Lymph node (LN) staging, especially preoperative mediastinal LN assessment, is an important assessment in lung cancer patients, with implications for prognosis as well as direct impacts on patient management.^[1]^ Positron emission tomography/integrated computed tomography (PET/CT) is considered as the best noninvasive staging modality in lung cancers,^[2,3]^ though the sensitivity and specificity of maximal standardized uptake values (SUV_max_) to predict LN malignancy have varied between studies. The sensitivity and specificity of the most widely used cutoff SUV_max_ of 2.5 were 40% to 97% and 60% to 96%, respectively.^[2–8]^ The reasons for this wide range included the study of different patient populations, varying criteria for malignancy, and patient-, tumor-, and technique-specific factors.^[9,10]^

Three previous studies reported on the use of the ratio of LN SUV_max_ to primary tumor SUV_max_ (SUV_n/t_), rather than SUV_max_, to predict mediastinal LN malignancy, with the aim of reducing the influence of tumor- and technique-specific factors on the diagnosis.^[11–13]^ Although those studies determined SUV_n/t_ to be a good predictor of mediastinal LN malignancy, the accuracies varied between studies, and tumor diameter, as an important indicator of LN metastasis, was not accounted for in the formula. Furthermore, most LNs in those studies were acquired by biopsy or mediastinoscopy, which is associated with a high potential false-negative rate. In Mattes et al's study,^[13]^ biopsy LNs were not restricted to LNs that appeared malignant on imaging, but were sampled at the discretion of the clinician who performed the biopsy. A total of 77% (392/504) of LNs were obtained by mediastinoscopy or fine needle aspiration, suggesting that up to 77% of PET/CT-positive LNs were not matched to their pathological diagnosis. Mediastinoscopy or fine needle aspiration were used to obtain 21.3% of LNs in Iskender et al's report, but the equivalent rate is not mentioned in Cerfolio's report.

We evaluated the relative capacities of SUV_n/t_, SUV_max_, and also SUV_n/t_ multiplied by maximal tumor diameter (SUV_index_) to predict mediastinal LN malignancy using LNs obtained by systemic lymph node resection to reduce the potential false-negative rate. We also evaluated the abilities of these parameters to predict LN malignancy among smokers and nonsmokers, respectively.

## 2 Methods

### 2.1 Ethics statement

This retrospective analysis was approved by the institutional review board of The Chinese People's Liberation Army (PLA) General Hospital. All data were extracted from the hospital medical database and were only used for academic research.

### 2.2 Study population

In this single-center retrospective study, we assessed 170 mediastinal LN stations from 73 patients who underwent fluorodeoxyglucose (FDG) PET/CT and thoracotomy with systemic LN resection between April 2008 and April 2016. A total of 895 consecutive patients underwent PET/CT because of suspicious lung cancer nodules at the Chinese PLA General Hospital, among whom 219 underwent surgery for highly suspected lung cancers. Patients were included if they had at least one detectable mediastinal LN on PET/CT with a LN SUV_max_ of 2.0 to 7.0 and an SUV_max_ value for suspicious primary lung cancer >2.0. The LN SUV_max_ was defined by LN station rather than by single LN because it was difficult to distinguish which LN within the station was responsible for the SUV_max_. We also assumed that the LN with the maximal SUV_max_ on PET/CT in each station was most likely to be the malignant one. All PET/CT-positive LN stations underwent systemic LN dissection.

Patients with a tumor history or who underwent chemotherapy before PET/CT scan were excluded to avoid additional effects on SUV_max_. Patients with ground glass opacity (GGO) lung cancer, multiple primary lung cancers, and patients whose mediastinal LNs were obtained via thoracotomy LN sampling, mediastinoscopy, and fine needle aspiration were also excluded to reduce the incidence of potential false-negatives.

Pathological results were matched to the PET/CT findings by LN station. We initially analyzed 441 mediastinal LN stations from 219 patients who had undergone both thoracotomy with systemic LN resection and PET/CT examination. Seventeen patients were excluded because their lung nodules turned out to be benign. A further 42 patients were excluded because all their mediastinal LNs were silent at PET/CT, 68 patients were excluded for having SUV_max_ <2.0 or >7.0, and 19 patients were excluded because of multiple primary lung cancers or GGO lung cancers.

### 2.3 PET/CT data collection and test methods

PET/CT scans were performed by using integrated PET/CT scanner (Siemens Biograph 64 High Definition). Patients were instructed to fast at least 6 hours before FDG administration. Whole body scans from the skull to feet (6 bed positions) were preformed 60 minutes later, after injection of 0.15 mCi/kg FDG. The CT examination performed for attenuation correction of PET images. Emission PET data were acquired for 2 minutes per bed position and iterative reconstruction with CT attenuation correction was performed. SUVmax of the primary and of each suspicious lymph node station was defined as the highest FDG uptake within a region of interest defined according to PERCIST criteria.^[14]^ Primary tumor largest dimension, primary tumor SUVmax, and LNs SUVmax were measured at the same time. We define SUVn/t as LN SUVmax divided by primary tumor SUVmax; SUVindex was defined as SUVn/t multiply by primary tumor largest dimension.

### 2.4 Statistical methods

Patient, tumor, and LN characteristics were reported as descriptive statistics. SUV_max_, SUV_n/t_, and SUV_index_ values were compared between pathologically positive and negative LNs using Mann–Whitney *U* tests. The areas under the curve (AUCs) for receiver-operating characteristic curves (ROC) were calculated for SUV_max_, SUV_n/t_, and SUV_index_ to assess their abilities to predict LN malignancy, with an optimal cutoff value for each parameter for all LNs. The optimal cutoff value was defined as the point on the ROC curve with the maximum sum of sensitivity and specificity. AUCs of the ROC curves were also used to evaluate the abilities of SUV_max_, SUV_n/t_, and SUV_index_ to predict malignancy in smokers and non-smokers, respectively. ROC curves were tested and compared using the Delong method.

Mann–Whitney *U* tests and ROC curves were tested and compared using Statistical Analysis System (SAS) version 9.4 (SAS Institute, Cary, NC). *P* values <0.05 were taken as significant.

## 3 Results

A total of 73 lung cancer patients (21 female, 52 male) with a mean age of 60.63 (median 60, range 36–81) years were included. Among all 73 patients, there were 170 PET/CT-positive mediastinal LN stations. All the LNs were confirmed pathology, with 28 malignant and 142 benign LN stations. The time interval between PET/CT and thoracotomy was 12.21 ± 5.56 (median 11, range 4–27) days. The tumor and patient characteristics are listed in Table 1 and the LN characteristics are listed in Table 2.

**Table 1:**
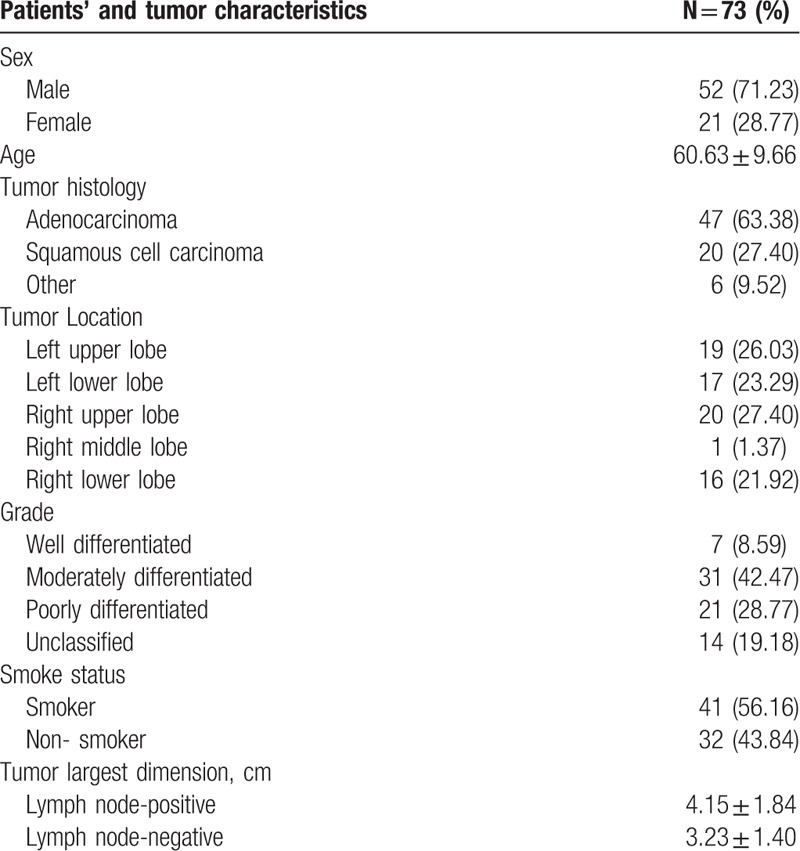
Patients’ and tumors’ characteristics.

**Table 2:**
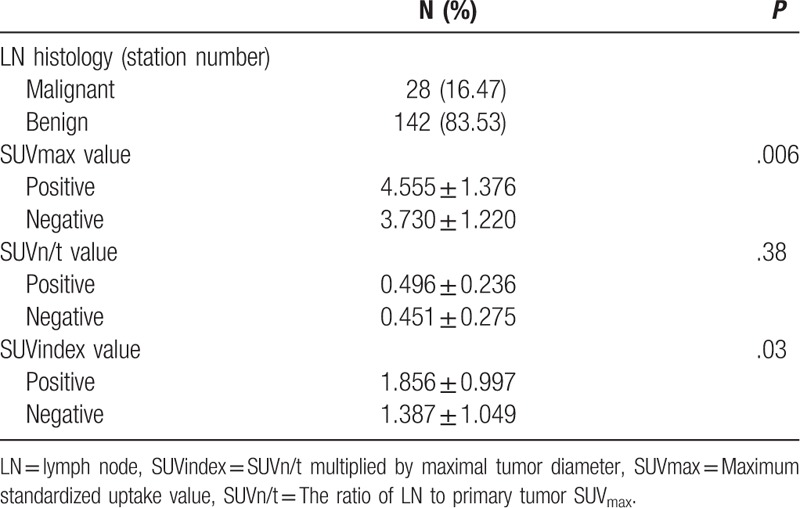
Lymph node characteristics.

**Figure 1:**
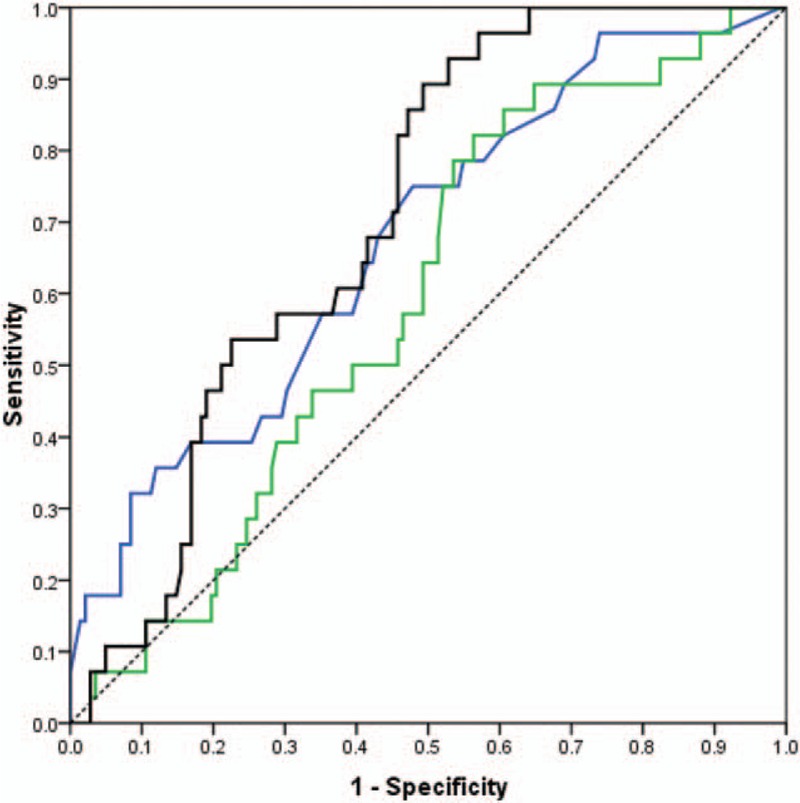
Receiver-operating characteristic (ROC) Curves for all patients who had both primary tumor SUVmax >2.0 and lymph nodes SUVmax 2.0 to 7.0, for SUVmax (blue), SUVn/t (green), and SUVindex (black) with diagonal reference line (dashed).

According to the ROC curves, SUV_index_ (AUC = 0.71, *P* < 0.001) was more accurate in predicting LN malignancy than SUV_max_ (AUC = 0.67, *P* < 0.001) and SUV_n/t_ (AUC = 0.59, *P* = 0.09) among all LNs (Fig. 1). The difference between SUV_index_ and SUV_n/t_ was statistically significant (*P* = 0.0245), but that between SUV_index_ and SUV_max_ was not significant (*P* = 0.60).

We also evaluated the diagnostic utilities of SUV_max_, SUV_n/t_, and SUV_index_ as predictors of LN malignancy among nonsmokers and smokers separately. SUV_index_ (AUC = 0.72, *P* < 0.001) was more accurate in predicting malignancy than SUV_max_ (AUC = 0.69, *P* < 0.001) and SUV_n/t_ (AUC = 0.62, *P* = 0.05) among non-smokers (LN stations = 75, N = 32). SUV_max_ (AUC = 0.71, *P* = 0.02) was more accurate than SUV_index_ (AUC = 0.69, *P* = 0.003) and SUV_n/t_ (AUC = 0.53, *P* = 0.84) in predicting LN malignancy among smokers (LN stations = 95, patients = 41). However, the differences between smokers and nonsmokers for all tests were not significant (*P* > 0.05).

**Figure 2:**
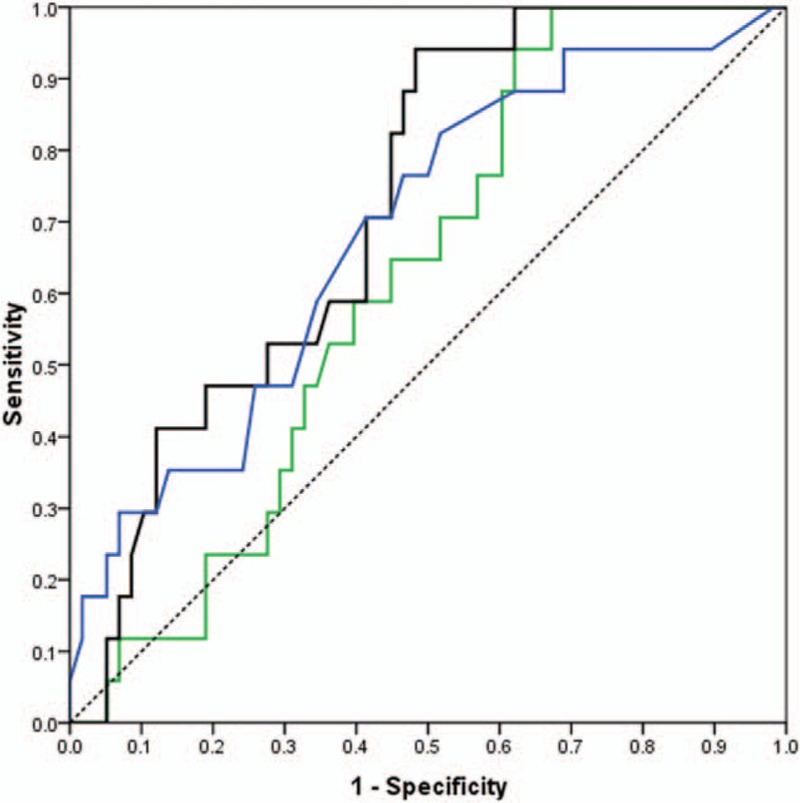
Receiver-operating characteristic (ROC) curves for non-smoker patients who had both primary tumor SUVmax >2.0 and lymph nodes SUVmax 2.0 to 7.0, for SUVmax (blue), SUVn/t (green), and SUVindex (black) With diagonal reference line (dashed).

**Figure 3:**
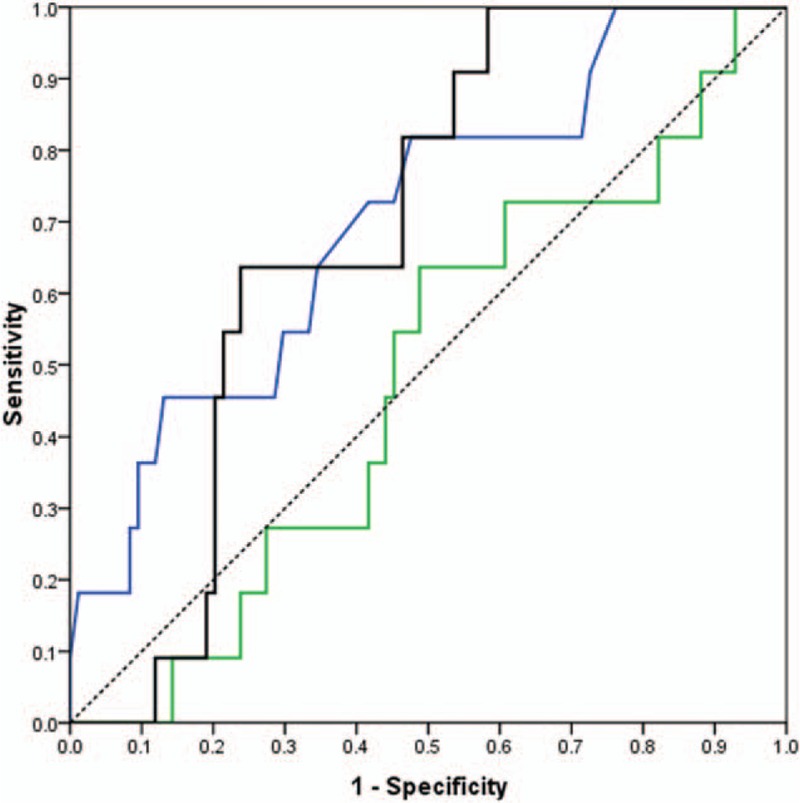
Receiver-operating characteristic (ROC) curves for smoker patients who had both primary tumor SUVmax >2.0 and lymph nodes SUVmax 2.0 to 7.0, for SUVmax (blue), SUVn/t (green), and SUVindex (black) with diagonal reference line (dashed).

**Figure 4:**
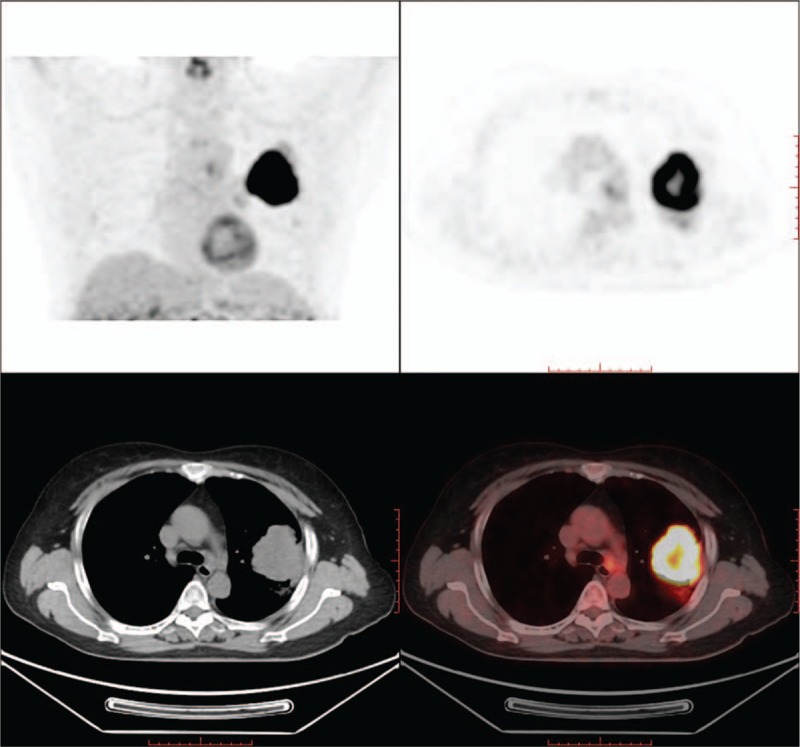
Nonsmoker lung cancer patients positron emission tomography/integrated computed tomography figure. Mediastinal lymph nodes SUVmax value was 2.2 and tumor SUVmax value was 23.9. This positron emission tomography/integrated computed tomography positive mediastinal lymph nodes turns out to be pathological benign.

**Figure 5:**
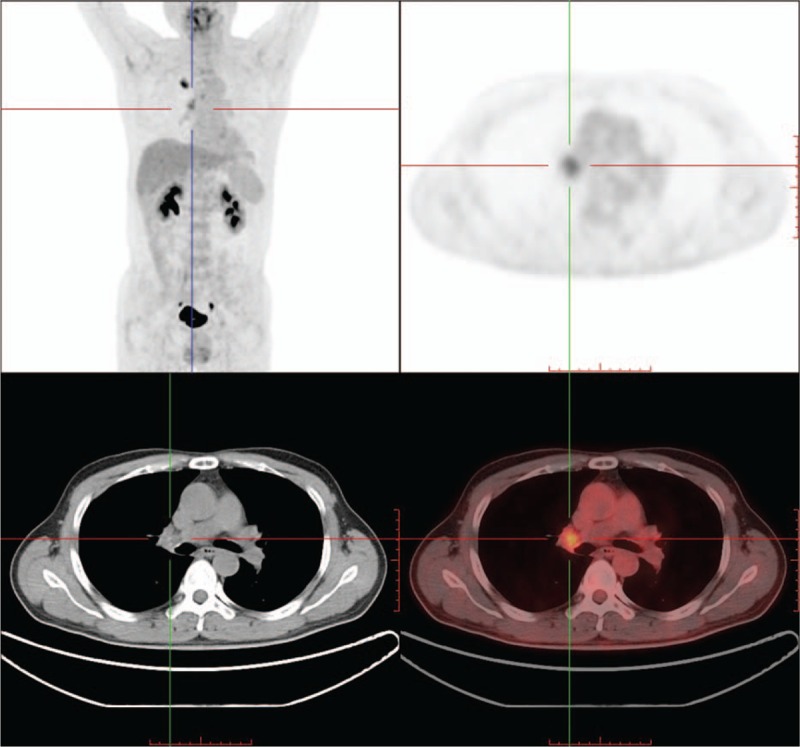
Smoker lung cancer patients positron emission tomography/integrated computed tomography figure. Mediastinal lymph nodes SUVmax value was 2.4 and tumor SUVmax value was 7.9. This positron emission tomography/integrated computed tomography positive mediastinal lymph nodes turns out to be pathological benign.

All the AUC values and 95% confidence intervals (CI), optimal cutoff values, sensitivities and specificities, and *P* values are listed in Table 3. ROC curves for smokers and nonsmokers are shown in Figures 2 and 3, and LN PET/CT images for nonsmokers and smokers are shown in Figures 4 and 5.

**Table 3:**
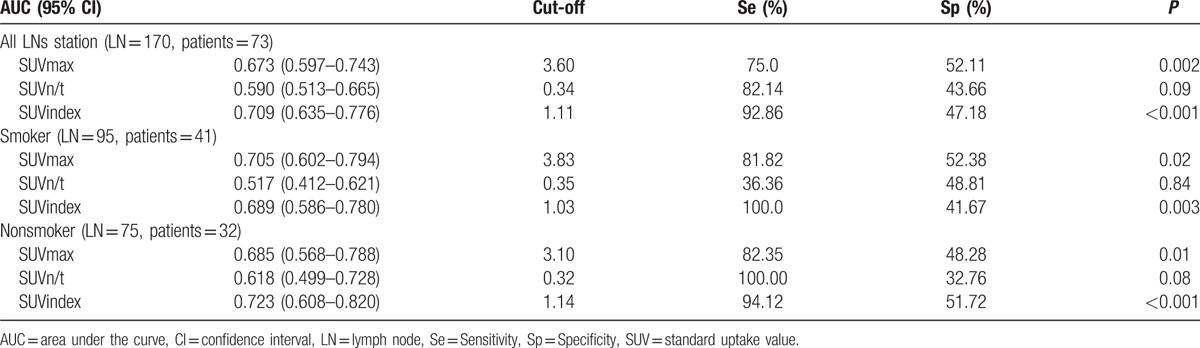
Receiver=operating characteristic curves parameters for different LN groups.

## 4 Discussion

SUV_max_ derived from PET/CT is the most widely used predictor of mediastinal LN malignancy in patients with lung cancer. However, the sensitivity and specificity using a cutoff value of 2.5 have varied considerably among studies. Differences may be caused by several factors, such as differences in patient populations, FDG blood levels, and tumor- and technique-specific factors, all of which may affect the abilities of SUV_max_ to diagnose LN malignancy accurately. We assessed the ability of SUV_n/t_, defined as lymph node SUV_max_ divided by tumor SUV_max_, to predict LN malignancy, to eliminate the impacts of some of these factors and thus standardize SUV_max_.

Three previous studies focused on the diagnostic accuracy of SUV_n/t_.^[11–13]^ Cerfolio and Bryant^[11]^ first confirmed SUV_n/t_ as a potential predictor of mediastinal LN malignancy in 2007. They assessed SUV_n/t_ in 335 FDG-avid mediastinal LNs in 239 patients, with an optimal cut-off for predicting malignancy of 0.56 (sensitivity 94%, specificity 72%) and AUC value of 0.79 (95% CI 0.66–0.88, *P* < 0.001). However, they did not consider the diagnostic utility of SUV_max_ or compare it with SUV_n/t_. Iskender's study in 2011 produced similar results. The AUC of SUV_n/t_ (referred to as PET predictive ratio) was 0.69 with an optimal cut-off of 0.49 for predicting malignancy (sensitivity 70%, specificity 65%).^[12]^ However, the diagnostic utility of LN SUV_max_ was assessed, with an optimal cutoff value 2.75 for predicting malignancy (sensitivity 84%, specificity 87%), whereas the AUC of SUV_max_ was not mentioned or compared with the PET predictive ratio. LN SUV_max_ was shown to be more accurate than SUV_n/t_ for predicting LN malignancy. The diagnostic utility of SUV_n/t_ in these 2 previous studies was similar to that found in our study, and SUV_max_ appeared to be more accurate than SUV_n/t_ for predicting mediastinal LN malignancy.

Mattes recently focused on the diagnostic utility of SUV_n/t_,^[13]^ using a similar study design to our present study. They also selected patients with both LN SUV_max_ values from 2.0 to 7.0 and primary tumor SUV_max_ values >2 because it was relatively difficult to distinguish if these LNs were malignant or benign based on SUV_max_ value alone. Mattes assessed and compared the diagnostic utilities of SUV_max_ and SUV_n/t_ and found that SUV_n/t_ (AUC 0.85, 95% CI 0.78–0.92) was significantly more accurate than SUV_max_ (AUC 0.65, 95% CI 0.55–0.76) for predicting LN malignancy. However, these results differed from those of the present study, which demonstrated that the accuracy of SUV_n/t_ (AUC 0.59, 95% CI 0.51–0.67) was worse than that of SUV_max_ (AUC 0.67, 95% CI 0.64–0.78).

Differences in the methods used to obtain the LNs may be the most important factor contributing to the differences in results between the present and Mattes's study. All LNs in the current study were obtained by systemic LN resection, whereas most of the LNs in previous studies were obtained by mediastinoscopic biopsy or thoracotomy LN sampling. LN biopsy via mediastinoscopy or sampling may lead to potential false-negative results because the biopsied LNs may not be the positive LNs displayed in the PET/CT image. This potential false-negative rate was mentioned as a major limitation in Mattes's study.^[13]^ Furthermore, most metastatic mediastinal LNs contain only microscopic tumor deposits, and it was therefore hard to obtain and confirm mediastinal LN tumor cells via mediastinoscopic biopsy or LN sampling. The false-negative rates of mediastinoscopic biopsy and LN sampling ranged from 4.4% to 8.2% according to previous studies.^[15–17]^ Differences between studies may also have arisen because we evaluated PET/CT-positive LNs by station, not by single positive LNs, as in all the previous studies. Furthermore, systemic LN resection guaranteed the resection of all PET/CT-positive LNs.

A third possible reason for the apparent discrepancies between study results may be the more-exclusive patient selection criteria employed in the present study. We excluded patients with GGO lung cancer because of its locally invasive characteristics and rare metastasis to LNs.^[18–21]^ We also excluded patients with multiple primary lung cancers because it was hard to confirm which tumors had caused the LN metastasis. However, unlike the previous studies, we did not exclude patients with small-cell lung cancer because we focused on the preoperative diagnostic accuracy of PET/CT, which was not limited to nonsmall cell lung cancers. It is not possible to perform preoperative lung nodule biopsies for all patients, and small-cell lung cancer patients may thus undergo surgery without pathologic confirmation, making it meaningless to limit the use of this diagnostic method to non-small cell lung cancer. Because the AUCs for SUV_n/t_ and SUV_max_ were not particularly informative, we hypothesized that tumor diameter may also influence the diagnostic accuracy because bigger or higher-stage tumors would have a greater possibility of LN metastasis.^[22–24]^ We therefore assessed the diagnostic efficiency of SUV_index_, defined as SUV_n/t_ multiplied by maximal tumor diameter on PET/CT. According to the results, SUV_index_ (AUC 0.71, 95% CI 0.64–0.78) provided a better predictive index of mediastinal LN malignancy in lung cancer patients than SUV_max_ or SUV_n/t_. The optimal SUV_index_ cutoff value was 1.11, with 92.86% sensitivity and 47.18% specificity. The SUV_index_ AUC was significantly higher than that for SUV_n/t_, though there was no significant difference in AUCs between SUV_max_ (AUC = 0.67, *P* = 0.002) and SUV_index_. However, it is possible that the lack of significance was because of the limited sample size in this study.

We also evaluated the AUCs for SUV_max_, SUV_n/t_, and SUV_index_ for predicting LN malignancy among non-smokers and smokers, respectively. Among smokers (LN station = 95, N = 41), SUV_max_ (AUC = 0.71) was more accurate for predicting LN malignancy than and SUV_n/t_ (AUC = 0.52) and SUV_index_ (AUC = 0.69), whereas SUV_index_ (AUC = 0.72) showed better diagnostic capabilities than SUV_n/t_ (AUC = 0.69) and SUV_max_ (AUC = 0.61) in nonsmokers. However, there were no significant differences among the 3 AUCs in either smokers or nonsmokers. It was interesting to note that the diagnostic abilities of SUV_max_ and SUV_index_ differed between smokers and nonsmokers, even though the differences were not statistically significant. The SUV_max_ and SUV_index_ ROC cutoff values in smokers were higher than in nonsmokers, indicating that malignant LNs had higher SUV_max_ values in smokers. This may be explained by an increase in noncarcinogenic SUV_max_ as a result of an inflammatory reaction in intrapulmonary LNs in smokers. These results suggest that it would be difficult to distinguish between inflammatory and malignant LNs on the basis of SUV_max_ value alone.^[25–27]^ In contrast, increases in LN SUV_max_ in nonsmokers are less likely to be caused by an inflammatory reaction, resulting in lower SUV_max_ values compared with smokers. However, given that there were no significant differences in AUCs for SUV_max_, SUV_n/t_, and SUV_index_ between smokers and nonsmokers, we were unable to conclude that SUV_index_ would be more accurate for predicting LN malignancy among nonsmokers than smokers.

All the patients in the current study underwent PET/CT and surgery in the same hospital. A lack of data from other hospitals was thus a major limitation of the present study, and we are unable to draw any conclusions about the abilities of SUV_index_ to predict LN malignancy in other centers, or using different PET/CT scanners. Our study was also limited by the relatively small sample size, as a result of excluding many patients to avoid potential false-positive LNs obtained by mediastinoscopic biopsy or LN sampling.

Further studies conducted in more centers and with larger sample sizes are needed to confirm the accuracy of SUV_index_ for predicting LN malignancy. Furthermore, the value of SUV_max_ for predicting malignancy has been reported to differ between LN stations, and further studies are needed to assess the predictive accuracy for each station.^[11]^

## 5 Conclusion

In conclusion, SUV_index_ may provide a more accurate preoperative prediction of mediastinal LN malignancy in patients with lung cancer patients compared with either SUV_max_ or SUV_n/t_.
